# Characteristics and outcomes of out-of-hospital cardiac arrest among students under school supervision in Japan: a descriptive epidemiological study (2008–2021)

**DOI:** 10.1265/ehpm.24-00319

**Published:** 2025-01-11

**Authors:** Kosuke Kiyohara, Mamoru Ayusawa, Masahiko Nitta, Takeichiro Sudo, Taku Iwami, Ken Nakata, Yuri Kitamura, Tetsuhisa Kitamura

**Affiliations:** 1 Department of Food Science, Faculty of Home Economics, Otsuma Women’s University, Tokyo, Japan; 2 Department of Nutrition and Health Science, Faculty of Health and Medical Science, Kanagawa Institute of Technology, Atsugi, Japan; 3 Department of Emergency Medicine, Osaka Medical and Pharmaceutical University, Osaka, Japan; 4 Department of Pediatrics, Osaka Medical and Pharmaceutical University, Osaka, Japan; 5 Division of Patient Safety, Osaka Medical and Pharmaceutical University Hospital, Osaka, Japan; 6 Institute of Human Culture Studies, Otsuma Women’s University, Tokyo, Japan; 7 Department of Preventive Services, Kyoto University School of Public Health, Kyoto, Japan; 8 Medicine for Sports and Performing Arts, Department of Health and Sports Sciences, Graduate School of Medicine, Osaka University, Suita, Japan; 9 Division of Environmental Medicine and Population Sciences, Department of Social and Environmental Medicine, Graduate School of Medicine, Osaka University, Suita, Japan

**Keywords:** Out-of-hospital cardiac arrest, School, Students, Japan, Etiology of arrest, Epidemiology

## Abstract

**Background:**

A comprehensive understanding of the epidemiology of pediatric out-of-hospital cardiac arrest (OHCA) occurring under school supervision is lacking. We aimed to comprehensively describe the characteristics and outcomes of OHCA among students in elementary schools, junior high schools, high schools, and technical colleges in Japan.

**Methods:**

OHCA data from 2008–2021 were obtained from the SPIRITS study, which provides a nationwide database of OHCAs occurring under school supervision across Japan. We included cases in which resuscitation was attempted by emergency medical service personnel or bystanders. The cases were classified into three groups based on their etiology: cardiac, non-cardiac, and traumatic origin. The primary outcome was one-month survival with favorable neurological outcomes, defined as a Glasgow–Pittsburgh cerebral performance category of 1 or 2. The demographic characteristics, event details, and outcomes were compared across the three groups by using χ^2^ tests for categorical variables and one-way analyses of variance for continuous variables.

**Results:**

During the 14-year study period, 602 OHCA cases were confirmed, with 430 (71.4%) classified as cardiac, 91 (15.1%) as non-cardiac, and 81 (13.5%) as traumatic origin. Non-cardiac and traumatic cases were less likely to be witnessed at the time of arrest (46.2% and 42.0%, respectively) than cardiac cases (82.6%; p < 0.001). Initiation of cardiopulmonary resuscitation by bystanders was less common in non-cardiac and traumatic cases (62.6% and 42.0%, respectively) than that in cardiac cases (82.8%; p < 0.001). The delivery of defibrillation using public-access automated external defibrillators was also significantly less frequent in non-cardiac (3.3%) and traumatic cases (6.2%) than that in cardiac cases (59.8%; p < 0.001). Ventricular fibrillation (VF) as the first documented rhythm was observed in 77.9% of cardiac cases but was much less common in non-cardiac (5.5%) and traumatic cases (8.6%; p < 0.001). One-month survival with favorable neurological outcomes was significantly lower in non-cardiac (6.6%) and traumatic cases (0%) than that in cardiac cases (50.2%; p < 0.001).

**Conclusions:**

OHCAs of cardiac origin were more frequently associated with VF and had relatively good prognoses. In contrast, OHCAs of non-cardiac and traumatic origins consistently resulted in poor outcomes, highlighting the critical importance of prevention strategies to reduce the occurrence of these incidents.

**Supplementary information:**

The online version contains supplementary material available at https://doi.org/10.1265/ehpm.24-00319.

## Graphical Abstract

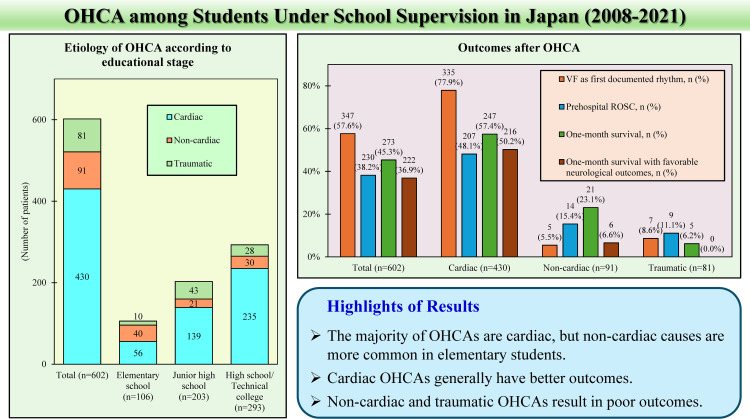

## Background

The occurrence of out-of-hospital cardiac arrest (OHCA) among schoolchildren in school settings is a tragic event with considerable social, communal, and familial impacts [1–4]. These events result in a marked loss of productive life years, substantial healthcare costs, and considerable emotional burden on families [4, 5], underscoring the importance of both prevention and outcome improvement. Understanding the specific characteristics of OHCA among schoolchildren is essential if effective intervention strategies are to be developed. However, the lack of systematic data collection in this setting has resulted in a scarcity of high-quality evidence worldwide [6].

To address this issue, we launched the Stop and Prevent Cardiac Arrest, Injury, and Trauma in Schools (SPIRITS) study in 2016. It was established to build a comprehensive registry of pediatric OHCAs occurring under school supervision across Japan toward the support of public-health research and development of effective prevention and intervention strategies. In 2018, we presented the initial results of the SPIRITS study, providing a descriptive epidemiological overview of OHCAs in school settings [7]. However, the study was limited by the relatively small number of cases available at the time, with an annual incidence of 30 to 50 cases and data spanning just six years. As a result, a comprehensive understanding of OHCA in these settings, particularly regarding the less common, non-cardiac causes, including trauma, was not fully achievable.

Continuous enrollment in the SPIRITS registry has allowed us to gather data over a period of 14 years, from 2008 to 2021, providing a robust foundation for more detailed analysis. This expanded dataset enabled us to conduct a more thorough, descriptive epidemiological study toward a better understanding of the characteristics and outcomes of OHCA among schoolchildren under school supervision. The objective of this study was to provide a detailed description of the characteristics, circumstances, and outcomes of pediatric OHCAs occurring under school supervision in Japan, with a specific focus on differences based on the etiology of the arrest.

## Methods

### Study design

This study was conducted as part of the SPIRITS study, a nationwide, prospective, observational study in which pediatric OHCA occurring under school supervision in Japan is examined. The rationale, design, and detailed methodology of the SPIRITS study have previously been described [7]. Briefly, it integrates data from two large-scale, national registries: the Injury and Accident Mutual Aid Benefit System of the Japan Sport Council (JSC) and the All-Japan Utstein Registry of the Fire and Disaster Management Agency (FDMA). The Injury and Accident Mutual Aid Benefit System records incidents of injury, illness, accidents, and deaths among students and younger children under school supervision, covering approximately 16 million students across Japan, with data on over 800,000 incidents reported annually [8]. The All-Japan Utstein Registry, adhering to the international Utstein format [9, 10], documents comprehensive data on OHCA cases managed by emergency medical service (EMS) personnel, including prehospital care and outcomes. The integration of these two databases enables a comprehensive analysis of pediatric OHCA cases in school settings across Japan.

### Study population

The study included all OHCAs that occurred among students in elementary schools (ages 6–12 years), junior high schools (ages 12–15 years), high schools (ages ≥15 years), and technical colleges (ages ≥15 years) under school supervision in Japan from April 1, 2008 to December 31, 2021. Cases were only included if resuscitation efforts were attempted by EMS personnel or if defibrillation was administered using an automated external defibrillator (AED) by bystanders, ensuring the inclusion of only those cases for which cardiac arrest was confirmed before hospital arrival. OHCAs among nursery-school children or kindergarteners were excluded, as their etiology and circumstances of arrest differ substantially from those of older students [11].

### Data collection

OHCA data for this study were extracted from the SPIRITS database. Variables collected included the following: demographic details, such as educational stage and sex; event characteristics, such as the date and time of the emergency call; the location of the arrest; the activity at the time of arrest; and whether the arrest was witnessed by a bystander or EMS. Clinical information included the origin of arrest, the first documented rhythm, the initiation of cardiopulmonary resuscitation (CPR) by a bystander, the usage of a public-access AED the administration of advanced airway management and epinephrine by EMS personnel, and survival outcomes after OHCA.

### Outcome measures

The primary outcome of the study was one-month survival with favorable neurological outcomes, defined as a Glasgow–Pittsburgh cerebral performance category (CPC) of 1 or 2 [9, 10]. Secondary outcomes were the incidence of ventricular fibrillation (VF) as the first documented rhythm, return of spontaneous circulation before hospital admission, and overall one-month survival after OHCA.

### Statistical analysis

The subjects were classified into three groups based on the etiology of arrest by using clinical records, EMS reports, and physician diagnoses: cardiac origin, non-cardiac origin, and traumatic origin. The demographic characteristics, event details, and outcomes were compared across the three groups by using χ^2^ tests for categorical variables and one-way analyses of variance for continuous variables. Statistical significance was set at a p-value of <0.05, ensuring that the results reflect statistically meaningful differences between the groups. All statistical analyses were conducted using SPSS version 27.0 J (IBM Corp., Armonk, NY, USA).

### Ethical considerations

This study was conducted in accordance with the principles outlined in the Declaration of Helsinki and was approved by the Ethics Committees of Otsuma Women’s University (approval number: 02-022) and Osaka University (approval number: 22007). Given the observational nature of the study and the use of de-identified data, the requirement for individual informed consent was waived by these committees.

## Results

### Study flow

**Fig. 1:**
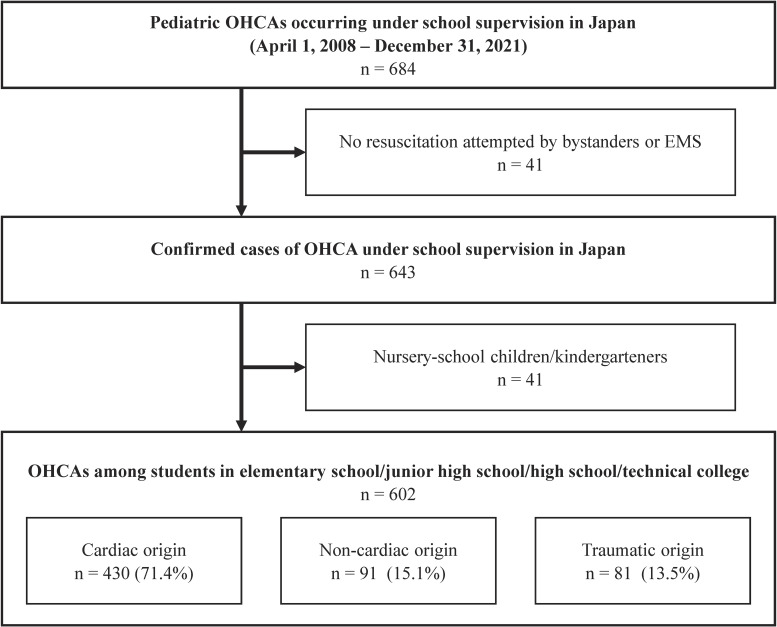
Study flow chart of OHCAs among Japanese students in this study.

Figure 1 shows the flow of pediatric OHCAs occurring under school supervision in Japan from April 1, 2008 to December 31, 2021 in this study. A total of 602 confirmed OHCA cases were included in the analysis. Of the 602 cases analyzed, 430 (71.4%) were classified as cardiac origin, 91 (15.1%) as non-cardiac origin, and 81 (13.5%) as traumatic origin.

### Origin of arrest

Table 1 shows the etiology of OHCA among the study sample. Cardiac-origin cases were largely due to idiopathic VF (n = 266) and hypertrophic cardiomyopathy (n = 30). Non-cardiac causes were primarily drowning (n = 26) and asphyxiation (n = 23), whereas the most common traumatic causes were falls (n = 32) and traffic accidents (n = 15). Cardiac causes were more frequent among high-school students (235/293, 80.2%), non-cardiac causes were more common in elementary-school students (40/106, 37.7%), and traumatic causes were most frequently observed among junior-high-school students (43/203, 21.2%). The details of etiology of OHCA according to educational stage and sex are provided in Additional File 1, showing that while the overall distribution of etiologies was similar between males and females, idiopathic VF was more frequently observed in males.

**Table 1:** Details of etiology of OHCA among Japanese students under school supervision according to educational stage

**Etiology of arrest**		**Educational stage**						**Total**	

		**Elementary ** **school**		**Junior high ** **school**		**High school/Technical college**			

		**n**	**(%)**	**n**	**(%)**	**n**	**(%)**	**n**	**(%)**
Cardiac (n = 430)	Idiopathic ventricular fibrillation	25	(23.6%)	82	(40.4%)	159	(54.3%)	266	(44.2%)
	Hypertrophic cardiomyopathy	1	(0.9%)	17	(8.4%)	12	(4.1%)	30	(5.0%)
	Commotio cordis	0	(0.0%)	5	(2.5%)	9	(3.1%)	14	(2.3%)
	Long QT syndrome	7	(6.6%)	1	(0.5%)	4	(1.4%)	12	(2.0%)
	Wolff–Parkinson–White syndrome	1	(0.9%)	2	(1.0%)	5	(1.7%)	8	(1.3%)
	Presumed cardiac (no definite diagnosis)	22	(20.8%)	32	(15.8%)	46	(15.7%)	100	(16.6%)

Non-cardiac (n = 91)	Drowning	11	(10.4%)	7	(3.4%)	8	(2.7%)	26	(4.3%)
	Asphyxiation	15	(14.2%)	3	(1.5%)	5	(1.7%)	23	(3.8%)
	Cerebrovascular disease	8	(7.5%)	4	(2.0%)	3	(1.0%)	15	(2.5%)
	Respiratory disease	2	(1.9%)	2	(1.0%)	6	(2.0%)	10	(1.7%)
	Aortic disease	0	(0.0%)	1	(0.5%)	3	(1.0%)	4	(0.7%)
	Other non-cardiac	4	(3.8%)	4	(2.0%)	5	(1.7%)	13	(2.2%)

Traumatic (n = 81)	Falls	3	(2.8%)	22	(10.8%)	7	(2.4%)	32	(5.3%)
	Traffic accidents	2	(1.9%)	6	(3.0%)	7	(2.4%)	15	(2.5%)
	Hanging	1	(0.9%)	10	(4.9%)	3	(1.0%)	14	(2.3%)
	Other external causes	4	(3.8%)	5	(2.5%)	11	(3.8%)	20	(3.3%)

Total		106		203		293		602	

The χ^2^ test revealed a significant difference in the distribution of etiologies by educational stage, with P < 0.001.

OHCA: Out-of-Hospital Cardiac Arrest

### Patient characteristics and circumstances at the time of arrest

As summarized in Table 2, the demographic characteristics and circumstances surrounding the OHCAs varied significantly across the groups. Non-cardiac cases occurred in younger students (mean age, 12.5 years) whereas patients with traumatic and cardiac etiologies had a mean age of 14.3 and 14.7 years, respectively (p < 0.001). Non-cardiac and traumatic cases predominantly occurred during non-exercise activities (75.8% and 84.0%, respectively), such as during classroom activities, commuting, or break times. Conversely, cardiac cases were more frequently linked to exercise-related activities (78.8%; p < 0.001). The location of the arrest also differed significantly among the groups. Traumatic OHCAs were more likely to occur outside school premises (59.3%), such as on the road or at home; non-cardiac and cardiac cases more commonly occurred within school premises (53.8% and 74.7%, respectively; p < 0.001). Additionally, a considerable proportion of traumatic cases (53.1%) were associated with suspected suicide attempts. These suspected suicide cases showed little sex difference, were predominantly observed among junior high school students (67.4%), and approximately 60% occurred outside school premises (Additional File 2).

**Table 2:** Patient characteristics and OHCA circumstances among Japanese students occurring under school supervision

	**Total**		**Etiology of arrest**						

			**Cardiac**		**Non-cardiac**		**Traumatic**		**P-value**
	**(n = 602)**		**(n = 430)**		**(n = 91)**		**(n = 81)**		
Males, n (%)	437	(72.6%)	329	(76.5%)	54	(59.3%)	54	(66.7%)	0.002
Age, years, mean (SD)	14.3	(2.9)	14.7	(2.6)	12.5	(3.7)	14.3	(2.4)	<0.001
Educational stage, n (%)									<0.001
Elementary school	106	(17.6%)	56	(13.0%)	40	(44.0%)	10	(12.3%)	
Junior high school	203	(33.7%)	139	(32.3%)	21	(23.1%)	43	(53.1%)	
High school/technical college	293	(48.7%)	235	(54.7%)	30	(33.0%)	28	(34.6%)	
Witness of arrest, n (%)									<0.001
Bystander witnessed	431	(71.6%)	355	(82.6%)	42	(46.2%)	34	(42.0%)	
EMS witnessed	35	(5.8%)	14	(3.3%)	12	(13.2%)	9	(11.1%)	
Not witnessed	136	(22.6%)	61	(14.2%)	37	(40.7%)	38	(46.9%)	
Location of arrest, n (%)									<0.001
Inside school premises (subtotal)	403	(66.9%)	321	(74.7%)	49	(53.8%)	33	(40.7%)	
Gymnasium	84	(14.0%)	80	(18.6%)	3	(3.3%)	1	(1.2%)	
Playground	193	(32.1%)	171	(39.8%)	7	(7.7%)	15	(18.5%)	
Pool	36	(6.0%)	28	(6.5%)	8	(8.8%)	0	(0.0%)	
Classroom	51	(8.5%)	20	(4.7%)	25	(27.5%)	6	(7.4%)	
Other	39	(6.5%)	22	(5.1%)	6	(6.6%)	11	(13.6%)	
Outside school premises (subtotal)	199	(33.1%)	109	(25.3%)	42	(46.2%)	48	(59.3%)	
Road	68	(11.3%)	45	(10.5%)	10	(11.0%)	13	(16.0%)	
Sports facility	42	(7.0%)	34	(7.9%)	5	(5.5%)	3	(3.7%)	
Home or dormitory	26	(4.3%)	7	(1.6%)	4	(4.4%)	15	(18.5%)	
Other	63	(10.5%)	23	(5.3%)	23	(25.3%)	17	(21.0%)	
Activity at the time of arrest, n (%)									<0.001
Exercise-related (subtotal)	374	(62.1%)	339	(78.8%)	22	(24.2%)	13	(16.0%)	
Extracurricular activities	174	(28.9%)	158	(36.7%)	7	(7.7%)	9	(11.1%)	
In class	157	(26.1%)	145	(33.7%)	9	(9.9%)	3	(3.7%)	
During break time	16	(2.7%)	13	(3.0%)	2	(2.2%)	1	(1.2%)	
Other school activities	27	(4.5%)	23	(5.3%)	4	(4.4%)	0	(0.0%)	
Non-exercise-related (subtotal)	228	(37.9%)	91	(21.2%)	69	(75.8%)	68	(84.0%)	
During break time	80	(13.3%)	37	(8.6%)	13	(14.3%)	30	(37.0%)	
Commuting to and from school	72	(12.0%)	27	(6.3%)	17	(18.7%)	28	(34.6%)	
Extracurricular activities	38	(6.3%)	11	(2.6%)	20	(22.0%)	7	(8.6%)	
In class	15	(2.5%)	7	(1.6%)	6	(6.6%)	2	(2.5%)	
Other school activities	23	(3.8%)	9	(2.1%)	13	(14.3%)	1	(1.2%)	
Suspected suicide attempt, n (%)	43	(7.1%)	0	(0.0%)	0	(0.0%)	43	(53.1%)	<0.001

EMS: Emergency Medical Service, OHCA: Out-of-Hospital Cardiac Arrest, SD: Standard Deviation

### Resuscitation by bystanders and EMS personnel

Table 3 presents the differences in resuscitation efforts across the three groups. Bystander-initiated CPR was performed in 82.8% of cardiac-origin cases, significantly higher than that in non-cardiac (62.6%) and traumatic cases (42.0%; p < 0.001). Defibrillation with an AED by bystanders was performed in 59.8% of cardiac-origin cases, which was notably higher than the 3.3% in non-cardiac cases and 6.2% in traumatic cases (p < 0.001). Non-cardiac and traumatic cases more frequently required advanced airway management and epinephrine administration by EMS personnel.

**Table 3:** Resuscitation by bystanders and EMS personnel of Japanese students experiencing OHCA under school supervision

	**Total**		**Etiology of arrest**						

			**Cardiac**		**Non-cardiac**		**Traumatic**		**P-values**
	**(n = 602)**		**(n = 430)**		**(n = 91)**		**(n = 81)**		
Bystanders’ response, n (%)									
AED delivery to the scene of arrest	395	(65.6%)	334	(77.7%)	38	(41.8%)	23	(28.4%)	<0.001
AED pad applied to patient’s chest	372	(61.8%)	317	(73.7%)	35	(38.5%)	20	(24.7%)	<0.001
Defibrillated via AED	265	(44.0%)	257	(59.8%)	3	(3.3%)	5	(6.2%)	<0.001
CPR performed									<0.001
Any type of CPR performed (subtotal)	447	(74.3%)	356	(82.8%)	57	(62.6%)	34	(42.0%)	
Chest compressions only	248	(41.2%)	191	(44.4%)	31	(34.1%)	26	(32.1%)	
Chest compressions + rescue breathing	199	(33.1%)	165	(38.4%)	26	(28.6%)	8	(9.9%)	
EMS response									
Advanced airway management, n (%)	115	(19.1%)	66	(15.3%)	26	(28.6%)	23	(28.4%)	0.001
Epinephrine administration, n (%)	70	(11.6%)	46	(10.7%)	14	(15.4%)	10	(12.3%)	0.443
Time from EMS call to contact with patient, min, mean (SD)	8.4	(3.6)	8.3	(3.3)	9.6	(4.7)	8.1	(3.4)	0.005
Time from EMS call to hospital arrival, min, mean (SD)	32.6	(14.8)	31.3	(13.2)	37.4	(19.3)	34.5	(16.1)	0.001

AED: Automated External Defibrillator, CPR: Cardiopulmonary Resuscitation, EMS: Emergency Medical Service, OHCA: Out-of-Hospital Cardiac Arrest, SD: Standard Deviation

### Outcomes

Table 4 summarizes the outcomes after OHCA, which varied markedly based on the etiology of the arrest. VF as the first documented rhythm was observed in 77.9% of cardiac-origin cases but was rare in non-cardiac (5.5%) and traumatic cases (8.6%; p < 0.001). One-month survival with favorable neurological outcomes (CPC 1 or 2) was higher in cardiac-origin cases (50.2%) and substantially lower in non-cardiac (6.6%) and traumatic cases (0%; p < 0.001). The outcomes after OHCA according to sex and the location of arrest are provided in Additional File 3. Overall, regardless of sex or location, non-cardiac and traumatic cases had significantly worse outcomes compared to cardiac-origin cases. Among these, males had better outcomes compared to females, and OHCAs occurring inside school premises showed better outcomes than those occurring outside.

**Table 4:** Outcomes after OHCA occurring among Japanese students under school supervision

**Outcomes**	**Total**		**Etiology of arrest**						

			**Cardiac**		**Non-cardiac**		**Traumatic**		**P-values**
	**(n = 602)**		**(n = 430)**		**(n = 91)**		**(n = 81)**		
VF as first documented rhythm, n (%)	347	(57.6%)	335	(77.9%)	5	(5.5%)	7	(8.6%)	<0.001
Prehospital return of spontaneous circulation, n (%)	230	(38.2%)	207	(48.1%)	14	(15.4%)	9	(11.1%)	<0.001
One-month survival, n (%)	273	(45.3%)	247	(57.4%)	21	(23.1%)	5	(6.2%)	<0.001
One-month survival with favorable neurological outcomes, n (%)	222	(36.9%)	216	(50.2%)	6	(6.6%)	0	(0.0%)	<0.001

OHCA: Out-of-Hospital Cardiac Arrest, VF: Ventricular Fibrillation

## Discussion

The present study provides a detailed epidemiological overview of OHCA among students under school supervision in Japan, using nationwide data collected over a 14-year period. Our study highlights significant differences in the characteristics, resuscitation efforts, and outcomes based on the etiology of the arrest, offering important insights that can guide future prevention and intervention strategies. Compared to our 2018 study [7], in which we analyzed only six years of data, this study extended the analysis period to 14 years, thereby more than doubling the number of cases. This expanded dataset has enabled the classification of OHCA cases among schoolchildren into three categories based on the etiology of the arrest, allowing for a detailed description of the characteristics and outcomes of each category. These insights are novel and valuable, contributing to a more comprehensive understanding of pediatric OHCA in schools. Furthermore, by restricting our analysis to confirmed OHCA cases in which resuscitation was attempted by EMS personnel, we ensured an accurate and rigorous description of OHCA occurrences and their outcomes, providing a clear picture of the epidemiological landscape in school settings.

Concerning cardiac-origin OHCAs, our results align with those of previous studies [7, 12–14], revealing that these cases are more commonly witnessed, more commonly involve bystander CPR or public-access defibrillation, and more commonly present with VF as the first rhythm than OHCAs with other etiologies. These factors likely contributed to the more favorable outcomes observed for this group, reflecting the success of intervention efforts in schools, such as the widespread availability of AEDs and the training of staff and students in CPR. Although these efforts have been effective, achieving the goals set forth by the Japanese Circulation Society’s “Aiming for Zero Deaths” initiative [15] will require continued efforts to further enhance emergency-response strategies in schools. Preventing OHCA with a cardiac etiology, however, remains challenging. Our study revealed that, whereas some cardiac arrests are linked to identifiable conditions such as hypertrophic cardiomyopathy, long QT syndrome, or Wolff–Parkinson–White syndrome, many occur without prior indicators, and even autopsies may not reveal a definitive cause [16–18]. This highlights the need for more effective school-based screening programs to identify at-risk individuals, although the prediction of cardiac events remains inherently difficult. Advances in testing and the development of more comprehensive screening protocols may offer potential pathways to better identification and prevention in the future.

Our results further indicated that OHCAs with non-cardiac and traumatic etiologies in school settings are less frequently witnessed and less commonly involve bystander interventions, such as CPR and AED use, than cardiac-origin OHCAs. However, compared to other locations, circumstances, and populations [19–21], the proportion of patients with non-cardiac or traumatic OHCA receiving these interventions in schools is still relatively high. The critical point is that, despite these efforts, the outcomes for non-cardiac and traumatic OHCA remain poor, similar to those in other settings [19–26]. This highlights the critical importance of prevention as the most effective strategy to reduce fatalities from these types of OHCAs. The Japan Resuscitation Council’s Resuscitation Guidelines 2020 also emphasize the need for further evidence regarding OHCAs with non-cardiac causes, such as trauma, drowning, and suicide [27]. Given that current guidelines highlight the lack of robust data, prevention strategies for such causes clearly remain underdeveloped, necessitating studies such as this one to inform future guidelines and improve preventive measures.

We want to highlight two important observations from our study. First, given that nearly half of the non-cardiac OHCAs are due to drowning or asphyxiation, the implementation of targeted prevention strategies is crucial. A recent technical report from the American Academy of Pediatrics emphasized that increased supervision during swimming activities and the presence of trained lifeguards significantly reduce the risk of drowning in school settings [28]. Additionally, regular safety education reportedly improves students’ preparedness to respond effectively if a fellow student is in danger of drowning [29, 30]. In Japan, the JSC has developed a manual for the prevention of swimming accidents in schools [31], and schools are expected to adhere to these guidelines. To prevent asphyxiation, students and staff must be educated about the risks of choking and ensuring vigilant monitoring during meals and other activities in which the risk of choking is high [32].

Second, the fact that more than half of traumatic OHCAs were linked to suspected suicide is alarming and underscores the urgent need for strong mental-health interventions in schools. Given that suicide is the leading cause of death among youth aged 10–19 years in Japan [33], schools play a crucial role in suicide prevention by providing a supportive environment [34]. The World Health Organization’s “LIVE LIFE” implementation guide for suicide prevention emphasizes the importance of fostering socio-emotional life skills, early identification of at-risk individuals, and providing immediate support for those with suicidal behaviors [35]. The implementation of comprehensive programs in schools that prioritize early detection, mental-health education, and accessible support systems is crucial to reduce the suicide risk and provide timely intervention for at-risk students [34, 35].

As shown in Table 1, the distribution of OHCA etiologies varies significantly by educational stage, highlighting the importance of implementing preventive strategies tailored to the specific needs of each group. Non-cardiac causes such as drowning and asphyxiation are more frequent among elementary school students, while cardiac-origin OHCAs are predominant in junior and senior high school students, with idiopathic VF being particularly common in high school students. Thus, for elementary school students, targeted interventions should include water safety education, close supervision during activities near water, and training for early recognition and management of choking incidents. For junior and senior high school students, the focus should shift toward early detection of cardiac conditions through school-based cardiac screening programs and ensuring the widespread availability of AEDs on school premises. Enhancing resuscitation education, including CPR and AED training for students and staff, is also essential to ensure preparedness for OHCA incidents and to improve survival outcomes. Additionally, across all educational stages, the consistent occurrence of OHCAs linked to suspected suicides underscores the critical need for robust mental health support systems.

To build evidence that contributes to the prevention of sudden deaths in school settings, future research should focus on evaluating the effectiveness of school-based cardiac screening programs, given the high occurrence of cardiac-origin OHCAs among students. Exploring barriers to bystander CPR and AED use, especially in non-cardiac and traumatic cases, is also important to improve survival rates. Furthermore, as environmental factors such as temperature and pollutants have been shown to have various impacts on health [36, 37], investigating their relationship with the occurrence of OHCAs in school settings could provide valuable insights for developing more effective preventive strategies.

### Limitations

This study has several limitations that should be acknowledged. First, although this study provides valuable descriptive data on pediatric OHCA in school settings, data could not be obtained for several critical factors. These include detailed information on the specific identities or roles of those who witnessed the cardiac arrest (e.g., teachers, staff, students, or visitors); the quality of bystander-initiated CPR; patients’ medical history and current medication; and specific details of in-hospital care, such as hemodynamic support, induced hypothermia, and coronary interventions. Additionally, we lacked data on environmental and situational factors, such as weather conditions at the time of arrest, the physical-activity level at the time of collapse, and the distribution and accessibility of public-access AEDs within school premises. The lack of this information limits the study’s ability to provide comprehensive guidance to improve prevention measures and post-arrest outcomes. Second, the etiology of many OHCA cases, particularly those classified as cardiac origin, was determined based on available data from the All-Japan Utstein Registry and the Injury and Accident Mutual Aid Benefit System. However, autopsies were not routinely performed, and across Japan, only 24,406 autopsies were conducted in 2022 [38]. This limitation introduces the possibility that some cases of underlying inherited cardiac conditions or electrical disorders were overlooked, potentially leading to misclassification or underestimation of certain causes of arrest. Third, the potential for input errors during the data-linkage process between the two national registries could have led to the underestimation or misclassification of OHCA cases [7]. Additionally, the exclusion of cases in which patients were not transported to hospitals by EMS personnel further limits the comprehensiveness of the incidence data, leading to a potential underreporting of the true occurrence and outcomes of OHCAs in school settings.

## Conclusions

This study offers a comprehensive analysis of pediatric OHCA occurring under school supervision in Japan, highlighting significant differences in characteristics, resuscitation efforts, and outcomes based on the etiology of arrest. Although patients experiencing OHCAs of cardiac origin often benefit from prompt intervention, resulting in relatively favorable survival rates, non-cardiac and traumatic OHCAs present a much grimmer outlook. Given that the majority of non-cardiac and traumatic OHCAs result in poor outcomes, the focus for such cases must increasingly prioritize robust prevention strategies.

## Abbreviations

AED: Automated External Defibrillator
CPC: Cerebral Performance Category
CPR: Cardiopulmonary Resuscitation
EMS: Emergency Medical Service
FDMA: Fire and Disaster Management Agency
JSC: Japan Sport Council
OHCA: Out-of-Hospital Cardiac Arrest
SPIRITS: Stop and Prevent Cardiac Arrest, Injury, and Trauma in Schools
VF: Ventricular Fibrillation

## Supplementary information

**Additional file 1:** **Supplementary Table 1.** Details of etiology of OHCA among Japanese students under school supervision according to educational stage and sex.

**Additional file 2:** **Supplementary Table 2.** Details of OHCA among Japanese students due to suspected suicide attempt.

**Additional file 3:** **Supplementary Table 3.** Outcomes after OHCA occurring among Japanese students under school supervision according to sex and location of arrest.

## Declarations

### Availability of data and material

Data cannot be shared for privacy or ethical reasons.
