# Supplementary material for: Characteristics and outcomes of out-of-hospital cardiac arrest among students under school supervision in Japan: a descriptive epidemiological study (2008–2021)
Source: Environ Health Prev Med. 2025 Jan 11;30:4. doi: 10.1265/ehpm.24-00319 (PMC11744026; doi:10.1265/ehpm.24-00319)
Supplement: Supplementary file 1 — Additional file 1: Supplementary Table 1. Details of etiology of OHCA among Japanese students under school supervision according to educational stage and sex. [file ehpm-30-004-s001.docx]

| **Supplementary Table 1. Details of etiology of OHCA among Japanese students under school supervision according to educational stage and sex** | | | | | | | | | | |
| --- | --- | --- | --- | --- | --- | --- | --- | --- | --- | --- |
| Sex | Etiology of arrest | | Educational stage | | | | | | Total | |
|  |  |  | Elementary school | | Junior high school | | High school/  Technical college | |  |  |
|  |  |  | n | (%) | n | (%) | n | (%) | n | (%) |
| Males  (P<0.001)* | Cardiac (n=329) | Idiopathic ventricular fibrillation | 11 | (20.8%) | 69 | (47.3%) | 132 | (55.5%) | 212 | (48.5%) |
|  |  | Hypertrophic cardiomyopathy | 0 | (0.0%) | 10 | (6.8%) | 8 | (3.4%) | 18 | (4.1%) |
|  |  | Commotio cordis | 0 | (0.0%) | 5 | (3.4%) | 9 | (3.8%) | 14 | (3.2%) |
|  |  | Long QT syndrome | 2 | (3.8%) | 0 | (0.0%) | 4 | (1.7%) | 6 | (1.4%) |
|  |  | Wolff–Parkinson–White syndrome | 1 | (1.9%) | 1 | (0.7%) | 5 | (2.1%) | 7 | (1.6%) |
|  |  | Presumed cardiac (no definite diagnosis) | 12 | (22.6%) | 24 | (16.4%) | 36 | (15.1%) | 72 | (16.5%) |
|  | Non-cardiac (n=54) | Drowning | 5 | (9.4%) | 4 | (2.7%) | 8 | (3.4%) | 17 | (3.9%) |
|  |  | Asphyxiation | 8 | (15.1%) | 3 | (2.1%) | 3 | (1.3%) | 14 | (3.2%) |
|  |  | Cerebrovascular disease | 4 | (7.5%) | 1 | (0.7%) | 3 | (1.3%) | 8 | (1.8%) |
|  |  | Respiratory disease | 1 | (1.9%) | 1 | (0.7%) | 3 | (1.3%) | 5 | (1.1%) |
|  |  | Aortic disease | 0 | (0.0%) | 0 | (0.0%) | 3 | (1.3%) | 3 | (0.7%) |
|  |  | Other non-cardiac | 2 | (3.8%) | 2 | (1.4%) | 3 | (1.3%) | 7 | (1.6%) |
|  | Traumatic (n=54) | Falls | 2 | (3.8%) | 10 | (6.8%) | 3 | (1.3%) | 15 | (3.4%) |
|  |  | Traffic accidents | 1 | (1.9%) | 5 | (3.4%) | 5 | (2.1%) | 11 | (2.5%) |
|  |  | Hanging | 1 | (1.9%) | 6 | (4.1%) | 2 | (0.8%) | 9 | (2.1%) |
|  |  | Other external causes | 3 | (5.7%) | 5 | (3.4%) | 11 | (4.6%) | 19 | (4.3%) |
|  | Total | | 53 | | 146 | | 238 | | 437 | |
| Females  (P<0.001)* | Cardiac (n=101) | Idiopathic ventricular fibrillation | 14 | (26.4%) | 13 | (22.8%) | 27 | (49.1%) | 54 | (32.7%) |
|  |  | Hypertrophic cardiomyopathy | 1 | (1.9%) | 7 | (12.3%) | 4 | (7.3%) | 12 | (7.3%) |
|  |  | Commotio cordis | 0 | (0.0%) | 0 | (0.0%) | 0 | (0.0%) | 0 | (0.0%) |
|  |  | Long QT syndrome | 5 | (9.4%) | 1 | (1.8%) | 0 | (0.0%) | 6 | (3.6%) |
|  |  | Wolff–Parkinson–White syndrome | 0 | (0.0%) | 1 | (1.8%) | 0 | (0.0%) | 1 | (0.6%) |
|  |  | Presumed cardiac (no definite diagnosis) | 10 | (18.9%) | 8 | (14.0%) | 10 | (18.2%) | 28 | (17.0%) |
|  | Non-cardiac (n=37) | Drowning | 6 | (11.3%) | 3 | (5.3%) | 0 | (0.0%) | 9 | (5.5%) |
|  |  | Asphyxiation | 7 | (13.2%) | 0 | (0.0%) | 2 | (3.6%) | 9 | (5.5%) |
|  |  | Cerebrovascular disease | 4 | (7.5%) | 3 | (5.3%) | 0 | (0.0%) | 7 | (4.2%) |
|  |  | Respiratory disease | 1 | (1.9%) | 1 | (1.8%) | 3 | (5.5%) | 5 | (3.0%) |
|  |  | Aortic disease | 0 | (0.0%) | 1 | (1.8%) | 0 | (0.0%) | 1 | (0.6%) |
|  |  | Other non-cardiac | 2 | (3.8%) | 2 | (3.5%) | 2 | (3.6%) | 6 | (3.6%) |
|  | Traumatic (n=27) | Falls | 1 | (1.9%) | 12 | (21.1%) | 4 | (7.3%) | 17 | (10.3%) |
|  |  | Traffic accidents | 1 | (1.9%) | 1 | (1.8%) | 2 | (3.6%) | 4 | (2.4%) |
|  |  | Hanging | 0 | (0.0%) | 4 | (7.0%) | 1 | (1.8%) | 5 | (3.0%) |
|  |  | Other external causes | 1 | (1.9%) | 0 | (0.0%) | 0 | (0.0%) | 1 | (0.6%) |
|  | Total | | 53 | | 57 | | 55 | | 165 | |

* The χ² test was used to examine differences in etiology according to educational stage

OHCA: Out-of-Hospital Cardiac Arrest
