# Supplementary material for: Characteristics and outcomes of out-of-hospital cardiac arrest among students under school supervision in Japan: a descriptive epidemiological study (2008–2021)
Source: Environ Health Prev Med. 2025 Jan 11;30:4. doi: 10.1265/ehpm.24-00319 (PMC11744026; doi:10.1265/ehpm.24-00319)
Supplement: Supplementary file 2 — Additional file 2: Supplementary Table 2. Details of OHCA among Japanese students due to suspected suicide attempt. [file ehpm-30-004-s002.docx]

| **Supplementary Table 2. Details of OHCA among Japanese students due to suspected suicide attempt** | | | |
| --- | --- | --- | --- |
| Total | | (n=43) | |
| Males, n (%) | | 22 | (51.2%) |
| Age, years, mean (SD) | | 14.5 | (2.7) |
| Educational stage, n (%) | |  |  |
|  | Elementary school | 4 | (9.3%) |
|  | Junior high school | 29 | (67.4%) |
|  | High school/technical college | 10 | (23.3%) |
| Etiology of arrest, n (%) | |  |  |
|  | Hanging | 14 | (32.6%) |
|  | Falls | 26 | (60.5%) |
|  | Electrocution | 1 | (2.3%) |
|  | Other external causes | 2 | (4.7%) |
| Location of arrest, n (%) | |  |  |
|  | Inside school premises (subtotal) | 17 | (39.5%) |
|  | Gymnasium | 1 | (2.3%) |
|  | Playground | 6 | (14.0%) |
|  | Classroom | 2 | (4.7%) |
|  | Other | 8 | (18.6%) |
|  | Outside school premises (subtotal) | 26 | (60.5%) |
|  | Road | 2 | (4.7%) |
|  | Home or dormitory | 14 | (32.6%) |
|  | Other | 10 | (23.3%) |

OHCA: Out-of-Hospital Cardiac Arrest, SD: Standard Deviation
