# Supplementary material for: Characteristics and outcomes of out-of-hospital cardiac arrest among students under school supervision in Japan: a descriptive epidemiological study (2008–2021)
Source: Environ Health Prev Med. 2025 Jan 11;30:4. doi: 10.1265/ehpm.24-00319 (PMC11744026; doi:10.1265/ehpm.24-00319)
Supplement: Supplementary file 3 — Additional file 3: Supplementary Table 3. Outcomes after OHCA occurring among Japanese students under school supervision according to sex and location of arrest. [file ehpm-30-004-s003.docx]

| **Supplementary Table 3. Outcomes after OHCA occurring among Japanese students under school supervision according to sex and location of arrest** | | | | | | | | | | | |
| --- | --- | --- | --- | --- | --- | --- | --- | --- | --- | --- | --- |
|  | | Outcomes | Total | | Etiology of arrest | | | | | | |
|  |  |  |  |  | Cardiac | | Non-cardiac | | Traumatic | | P-values |
| Sex | Males | VF as first documented rhythm, n (%) | 278 | (63.6%) | 269 | (81.8%) | 3 | (5.6%) | 6 | (11.1%) | <0.001 |
|  |  | Prehospital return of spontaneous circulation, n (%) | 179 | (41.0%) | 163 | (49.5%) | 10 | (18.5%) | 6 | (11.1%) | <0.001 |
|  |  | One-month survival, n (%) | 215 | (49.2%) | 197 | (59.9%) | 13 | (24.1%) | 5 | (9.3%) | <0.001 |
|  |  | One-month survival with favorable neurological outcomes, n (%) | 182 | (41.6%) | 177 | (53.8%) | 5 | (9.3%) | 0 | (0.0%) | <0.001 |
|  |  | Total | n=437 | | n=329 | | n=54 | | n=54 | |  |
|  | Females | VF as first documented rhythm, n (%) | 69 | (41.8%) | 66 | (65.3%) | 2 | (5.4%) | 1 | (3.7%) | <0.001 |
|  |  | Prehospital return of spontaneous circulation, n (%) | 51 | (30.9%) | 44 | (43.6%) | 4 | (10.8%) | 3 | (11.1%) | <0.001 |
|  |  | One-month survival, n (%) | 58 | (35.2%) | 50 | (49.5%) | 8 | (21.6%) | 0 | (0.0%) | <0.001 |
|  |  | One-month survival with favorable neurological outcomes, n (%) | 40 | (24.2%) | 39 | (38.6%) | 1 | (2.7%) | 0 | (0.0%) | <0.001 |
|  |  | Total | n=165 | | n=101 | | n=37 | | n=27 | |  |
| Location  of arrest | Inside school  premises | VF as first documented rhythm, n (%) | 270 | (67.0%) | 260 | (81.0%) | 5 | (10.2%) | 5 | (15.2%) | <0.001 |
|  |  | Prehospital return of spontaneous circulation, n (%) | 184 | (45.7%) | 167 | (52.0%) | 11 | (22.4%) | 6 | (18.2%) | <0.001 |
|  |  | One-month survival, n (%) | 219 | (54.3%) | 203 | (63.2%) | 14 | (28.6%) | 2 | (6.1%) | <0.001 |
|  |  | One-month survival with favorable neurological outcomes, n (%) | 183 | (45.4%) | 177 | (55.1%) | 6 | (12.2%) | 0 | (0.0%) | <0.001 |
|  |  | Total | n=403 | | n=321 | | n=49 | | n=33 | |  |
|  | Outside school  premises | VF as first documented rhythm, n (%) | 77 | (38.7%) | 75 | (68.8%) | 0 | (0.0%) | 2 | (4.2%) | <0.001 |
|  |  | Prehospital return of spontaneous circulation, n (%) | 46 | (23.1%) | 40 | (36.7%) | 3 | (7.1%) | 3 | (6.3%) | <0.001 |
|  |  | One-month survival, n (%) | 54 | (27.1%) | 44 | (40.4%) | 7 | (16.7%) | 3 | (6.3%) | <0.001 |
|  |  | One-month survival with favorable neurological outcomes, n (%) | 39 | (19.6%) | 39 | (35.8%) | 0 | (0.0%) | 0 | (0.0%) | <0.001 |
|  |  | Total | n=199 | | n=109 | | n=42 | | n=48 | |  |

OHCA: Out-of-Hospital Cardiac Arrest, VF: Ventricular Fibrillation
